# A vital physiological parameter mobile monitoring system for elderly patients in remote areas

**DOI:** 10.1186/s12911-026-03499-w

**Published:** 2026-05-16

**Authors:** Zhihui Shang, Jiamei Feng, Mengxi Xiao, Haixia Su, Jue Liu, Taotao Pan, Li Dong, Tao Mi, Ziqiang Wang

**Affiliations:** 1 College of Medical Information Engineering, Zunyi Medical University, Xinpu District, Zunyi, Guizhou 563006 China; 2 Comprehensive Health Data Research Institute, Zunyi Medical University, Xinpu District, Zunyi, Guizhou 563006 China; 3 College of Information Engineering, Henan University of Science and Technology, Kaiyuanlu District, Luoyang, Henan 471000 China; 4 School of Network Security, Jinling Institute of Technology, Jiangning District, Nanjing, Jiangsu 211169 China

**Keywords:** Remote monitoring, STM32, Sensor, MQTT protocol, Android application

## Abstract

**Background:**

The health monitoring situation for elderly patients in remote areas remains challenging. The use of modern computer technology, sensor technology, and mobile communication to provide real-time remote monitoring of vital physiological parameters for elderly patients, especially those with special medical conditions, is highly important.

**Objective:**

To provide portable and real-time remote monitoring of vital physiological parameters and to increase the level of health care for elderly patients in remote areas, this study designed and implemented an Android application-based remote vital physiological parameter monitoring system.

**Methods:**

By utilizing the STM32F103RCT6 microcontroller as the central processor and employing microcontroller technology, wireless communication, the MQTT protocol, sensor technology, and Android application development, a user-friendly health monitoring system that enables real-time vital physiological parameter monitoring, and location tracking capabilities is realized.

**Significance:**

This system can provide alerts for abnormal vital physiological data, effectively responding to sudden emergency health conditions. Additionally, it can serve as a daily tool for monitoring vital physiological parameters, helping to monitor the health of elderly patients in remote areas and improve their quality of life.

## Background

Population aging has exacerbated numerous social issues. For example, the majority of elderly people suffer from multiple chronic diseases and have poor self-care abilities, placing a great burden on the health care system. This increases the demand for health care professionals and leads to a continuous increase in medical expenses for patients and their families. The level of medical care of health care systems must be improved to cope with growing clinical pressures. The requirements for medical technology have surpassed the pace of scientific and technological advancements [1, 2].

Furthermore, owing to fast-paced lifestyles, high work pressures, and a lack of exercise, various chronic diseases are becoming more prevalent among younger populations. This imposes a substantial economic burden on individuals, families, and society and places greater demands on the current health care model. These challenges are driving health care reform and shifting the medical model from a traditional approach of hospital-based diagnosis, disease detection, and subsequent treatment to preventive, early detection, and early treatment. Health check-ups and health monitoring play crucial roles in the new health care model. With the ongoing development of digitalization in society, there is an increasing demand for health monitoring among the population. Therefore, the development of remote health monitoring devices has garnered significant attention.

The development of sensor technology, computer technology, and mobile communication technology has facilitated recent progress in remote monitoring [3]. In remote health monitoring, sensors that measure vital signs, such as heart rate, pulse, oxygen, and blood pressure sensors, have been continuously upgraded, enabling rapid development in remote health monitoring. Remote health monitoring enables doctors to monitor patients’ health conditions in real time from a remote location, effectively preventing emergencies and providing timely aid during critical moments. Current related systems demonstrate tremendous potential, particularly for elderly patients in remote areas with limited medical resources and physically disabled patient populations. In recent years, there have been numerous studies and applications in remote health monitoring [4–8]. We review existing researches in three aspects: (1) research on healthcare-related monitoring systems, (2) research related to health parameter monitoring, and (3) research on health monitoring for elderly individuals.

Numerous studies emerged in the field of healthcare-related monitoring systems. In response to the rapid increase in the aging population and the challenges it poses in health care and social care, Al-Khafajiy proposed an intelligent medical monitoring system. The system is designed to assist elderly patients and people with disabilities in living independently while continuously monitoring their health conditions, and alleviating concerns regarding emergency situations or life-threatening health conditions [9]. Tseng developed an intelligent health monitoring system based on nursing homes that alerts health care providers or caregivers in emergency situations [10]. Yu proposed an integrated health monitoring system to address the health care monitoring needs of elderly patients, offering computer-assisted decision support for clinical physicians and community nurses [11]. However, the system lacks remote physician verification of predictive vital parameters and cannot notify health care providers in emergency situations.

Similarly, there is a considerable amount of research related to health parameter monitoring. Avnish S. J. proposed a smartwatch for potential dementia patients. Their system uses sensors in the smartwatch to collect vital sign data but does not involve remote access by health care professionals [12]. Maria introduced a non-invasive heart rate monitoring system using a PPG heart rate monitoring sensor and an OLED fingertip SpO2 sensor. However, the measured data from this system have not been validated by any medical practitioners or published research [13]. Ammar A. M. developed a multiparameter monitoring system that tracks five physiological parameters: heart rate, noninvasive blood pressure, temperature, electrocardiogram, and blood oxygen saturation [14]. Alessio developed a health management system for patients with diabetes. The device tracks food intake, blood pressure, insulin levels, caloric intake, medications, and activities [15]. Kavitha introduced a portable physical monitoring framework that periodically monitors patients’ heart rate, body temperature, and other vital signs [16]. ABM Rezbaul Islam addressed the global public health challenge of depression and stressed burden on pregnant women during and after pregnancy by implementing continuous remote monitoring of users’ mental health status through a smartphone application [17]. Additionally, psychological education interventions by trained community health care workers may help minimize this public health crisis to a great extent. To enable users to enjoy personalized and networked real-time health monitoring, Weichao designed an intelligent monitoring system [18]. This system not only feeds various collected physiological data back to smart devices via wireless mode but also provides data services to home monitoring terminals of the community medical system. Ammireddy developed a health monitoring system that uses the IoT to monitor the health parameters of pregnant women and infants [19]. The device can monitor blood pressure, pulse, body temperature, oxygen levels and obtain electrocardiogram. Neha developed an artificial intelligence-based electronic health monitoring system for monitoring patient parameters [20]. The system integrates networking, mobile, and display technologies. In the proposed system, temperature, pulse rate, blood pressure, and blood glucose levels are captured and stored in a database. S. Arun Kumar implemented an intelligent health monitoring system that combines machine learning and IoT technologies [21]. The system comprises temperature sensors, pulse oximeter sensors (for collecting heart rate and oxygen saturation), and a blood pressure sensing module to track patients’ health status. S. Nagaraj developed an energy-efficient, reliable, cost-effective, and user-friendly patient monitoring system [22]. This system efficiently collects and reports real-time patient-related data, enabling physicians and other health care assistants to remotely monitor the patients’ health parameters such as temperature, heart rate, electrocardiography, and location. Furthermore, the system operates in a bidirectional process, allowing health care professionals to not only provide assistance but also empower caregivers to offer suggestions they deem necessary. Shailesh proposed an IoT-based smart health monitoring system to improve the clinical care of cardiac patients [23]. By continuously monitoring heart rate, oxygen concentration, and body temperature parameters through 24-hour monitoring with heartbeat sensors, temperature sensors, and SpO2 sensors, the system can promptly provide medication when changes in heart rate, oxygen levels, and temperature occur.

In the field of health monitoring for elderly individuals, Mondher proposed a detection method based on an infrared array sensor to detect the presence of people in a room for monitoring elderly patients living alone [24]. They utilized a wide-angle low-resolution sensor (specifically, pixels) to collect thermal information from the monitoring area and employed deep learning to recognize the presence of up to three individuals with an accuracy of 97$\%$. Their approach also achieved 100$\%$ accuracy in detecting the presence or absence of individuals. Chandler J designed multiple remote vital sign estimation methods, forming a multifunctional remote health monitoring system [25]. The system includes (1) a camera-based system to estimate the patient’s heart rate, blood pressure, SpO2, and body temperature under the assumption that sufficient light is present and (2) a radar-based method for estimating the patient’s heart rate and respiratory rate. Mostafa proposed a solution for remote respiration and sleep position monitoring using radar [26]. The system can monitor multiple individuals simultaneously, effectively increasing the number of targets detected and reducing costs by minimizing the number of sensors. Additionally, the system can identify the sleep positions of each monitored individual by selecting appropriate target features and using a support vector machine (SVM) classifier. Compared with the reference sensors, the monitoring subsystem achieved an accuracy of over 97$\%$ for monitoring the respiratory parameters of human subjects in the bedroom and an accuracy of over 83$\%$ for sleep position detection. Wang Yue described the development of a software and hardware system for monitoring the health status of vehicle drivers [27]. The system uses portable devices to monitor human physiological parameters and transmits the data to mobile terminals or uploads it to cloud servers for data analysis. Tuyisenge proposed a system for monitoring the health of infants in which an intelligent restaurant was added to the existing solution by using cameras, fingerprint-based access, indoor temperature, and humidity control through a fuzzy inference system [28]. The system also includes an alert system that reminds a caregiver to feed the baby. If the caregiver fails to comply with the feeding time, an alarm is sent to the parents to remind the caregiver to do so.

Health monitoring for elderly patients is very important, especially for elderly patients who live alone in remote areas with limited medical resources. Yu et al. [29] developed an intelligent elderly care robot based on a robot operating system. This robot is capable of patrolling within a designated area, monitoring the status of the elderly and potential safety risks, collecting physiological data from elderly individuals, and uploading these data in real time to an IoT platform to be shared with family members or caregivers. To promptly detect and assist in the event of an elderly person falling, as well as to monitor their vital signs in real time, Shen et al. [30] designed a health monitoring and fall prevention positioning alarm device for elderly individuals. This device integrates sensors for monitoring body temperature, heart rate, step count, etc., to continuously monitor the health data of elderly individuals. It quickly raises an alarm when the data indicate any abnormalities. Hosseinzadeh et al. [31] proposed an IoT-based health monitoring system that uses smart elderly care technology to check vital signs and detect biological and behavioral changes. It provides a health monitoring system for the involved medical team, which uses sensor technology through IoT devices to continuously monitor and evaluate the behavioral activities and biometric parameters of disabled individuals or elderly individuals.

On the basis of the aforementioned studies, remote health monitoring can be used in many scenarios, such as for elderly people, disabled people, and people with specific diseases. These systems can be used for in-hospital monitoring, daily home monitoring, and monitoring in nursing homes. Thus, remote health monitoring systems have strong customization properties, and different systems exist in the market to meet the needs of different users. However, their quality varies. Traditional medical devices often have disadvantages such as large size, high cost, and lack of portability, so they are not suitable for the main user group of elderly individuals. Most existing health monitoring systems are based on Bluetooth communication nodes that must be paired with smart terminals, which limits the mobility of home monitoring terminals and increases the system cost. Remote monitoring requires that the distance be ignored and that the user’s physical condition be monitored in real time. This requires the monitoring device to be small in size, easy to carry, simple to use, capable of real-time monitoring and reliable in terms of transmission. Owning to differences in sensor performance, current related systems do not meet these requirements.

To provide elderly patients residing in remote areas with limited resource access to a portable, real-time, dependable, and economically viable system for remote monitoring of vital physiological parameters, this study designs and implements a small, portable and user-friendly remote health monitoring system based on an Android application.

The contributions of this project are as follows:

1. Multisensor integration: A health monitoring system with features such as energy efficiency, reliability, cost effectiveness, convenience, and real-time monitoring of vital physiological parameters and location tracking is implemented.
2. Real-time monitoring: An STM32-based health monitoring application is developed that can remotely monitor vital physiological data in real time, with a wide range of usage scenarios.
3. Timely alerts: The system supports users in customizing the threshold of physiological parameters, forming a personalized health monitoring system and providing timely warnings in abnormal situations.

## Methods

### Overall design

Prior to discussing the detailed research methods, we define the key technical terms in Table 1 to allow for a clearer interpretation of this paper.

**Table 1:** Glossary of terms

Term	Full Name	Explanation
MQTT	Message Queuing Telemetry Transport	A lightweight messaging protocol designed for small sensors and mobile devices, optimized for high-latency or unreliable networks and commonly used in IoT applications for efficient data transmission.
PPG	Photoplethysmography	A noninvasive optical technique used to detect blood volume changes in the microvascular bed of tissue. It is commonly used to measure heart rate and blood oxygen saturation (SpO2).
GNSS	Global Navigation Satellite System	A general term describing any satellite navigation system that provides geolocation and time information to a GPS receiver anywhere on or near the Earth where there is an unobstructed line of sight to four or more satellites. Examples include GPS, GLONASS, Galileo, and Beidou.
UART	Universal Asynchronous Receiver Transmitter	A hardware device used for serial communication, converting parallel data to serial data and vice versa. It is widely used in embedded systems for communication between microcontrollers and other devices.
USART	Universal Synchronous/Asynchronous Receiver Transmitter	An advanced version of UART that supports both synchronous and asynchronous communication, allowing for more flexible data transfer options in embedded systems.
NB	Narrowband	Communication technology that uses a narrow bandwidth, typically used in IoT and M2M applications where low power consumption and long-range communication are needed. Examples include Narrowband Internet of Things (NB-IoT).
PWM	Pulse Width Modulation	A technique used to control the average value of voltage (or current) supplied to a load by varying the duty cycle of a square wave signal. It is commonly used in motor control, LED dimming, and audio applications to regulate power delivery efficiently.

**Fig. 1:**
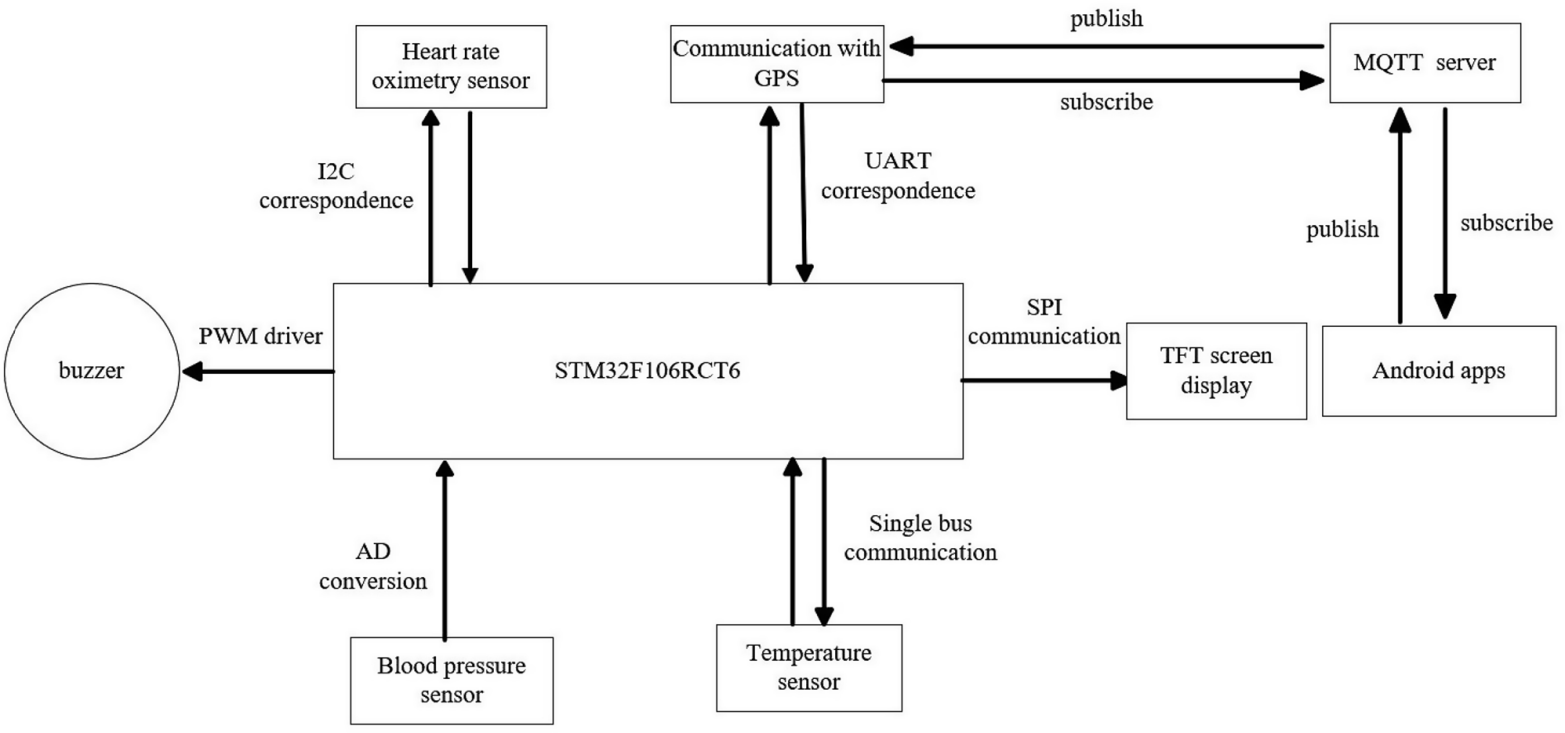
Overall structural framework of the system

The remote health monitoring system designed in this study targets elderly patients in remote areas with limited medical resources and is also suitable for the general public who need to monitor their own physiological data. To cater to the diverse user base, in addition to deploying hardware components, a location feature is specifically incorporated into the mobile software. This enables the received latitude and longitude data to be converted into actual physical addresses and displayed on a map, providing users with a more intuitive visualization of the device’s location. On the basis of earlier requirements analysis, the designed system consists of eight modules: a control module, a communication and GPS module, a heart rate and blood oxygen detection module, a blood pressure detection module, a body temperature detection module, a display module, a warning module, and a mobile terminal module. The overall system architecture is illustrated in Fig. 1.

The control module uses the MCU STM32F103RCT6 minimum system to control the whole system. The controller uses the ARM Cortex-M3 core, which has high performance and relatively low cost. The BC20 module is used for communication and the GPS module, which integrates two functions - NB-IoT and GNSS can obtain latitude and longitude and achieve wireless communication. Moreover, it has the advantages of low cost, low power consumption, high performance, wide coverage of cellular data connections, and stable connections, and the drive method is simple. A MAX30102 sensor is used for heart rate and blood oxygen detection. Through red light and infrared light, human physiological data can be collected by the sensor. Pulse and blood oxygen saturation are measured using blood vessel beating when the body tissue generates a transmittance difference. The blood pressure detection module uses an MPS20 pressure sensor to simulate blood pressure collection. The human body temperature detection module uses the DS18B20 temperature sensing module, which can measure the temperature. The display module uses a 1.44 inch TFT color screen to meet achieve a clear and unambiguous display. The early warning module uses a passive buzzer to remember when the human data are abnormal. Finally, the mobile terminal, on the mobile phone software shows the physiological data of the device user at any time. A map displays the location of the device, so that the guardian can quickly find the location of the patient and address emergencies.

### Hardware design

**Fig. 2:**
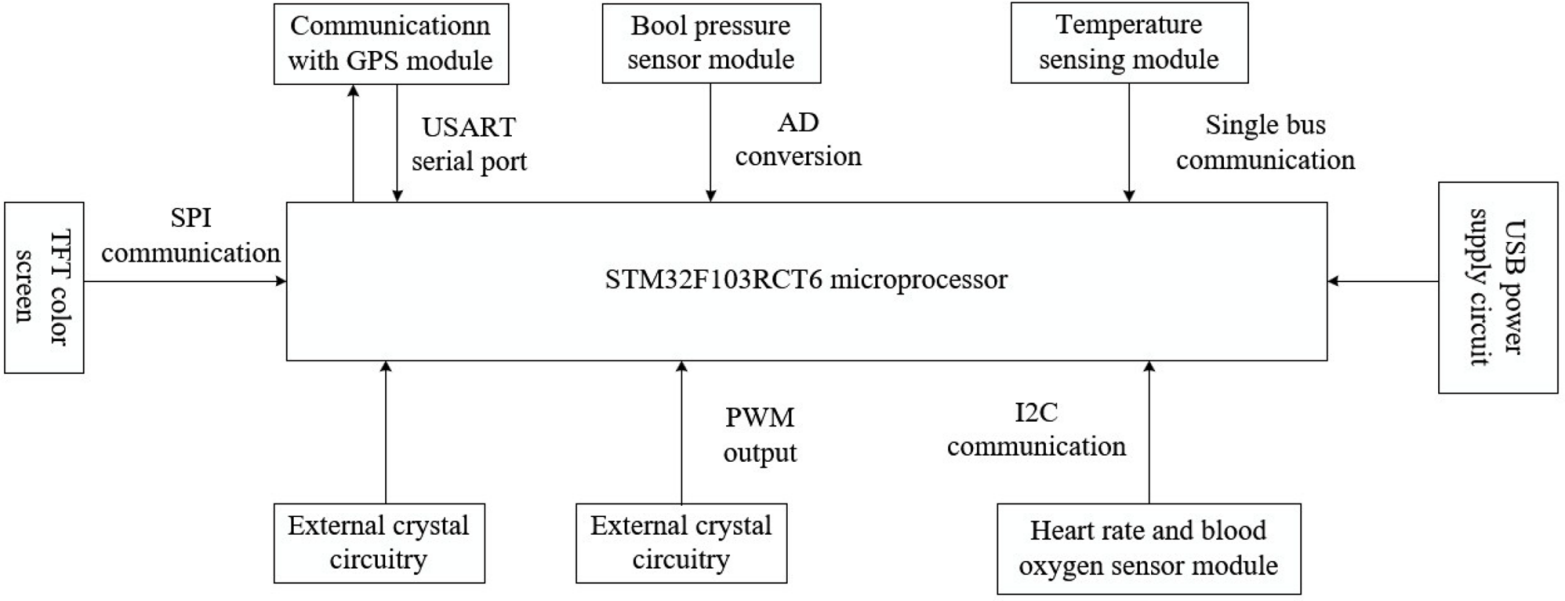
Overall hardware structure of the system

The overall hardware structure of the system is shown in Fig. 2, it consists of a microprocessor module, a remote communication and GPS module, a sensor module (heart rate, blood oxygen, blood pressure and temperature sensors), an early warning module, and a display module. These modules can be divided into four parts: a control unit, an information-receiving and information-sending unit, a data acquisition unit, and an early warning and display. Each part works in coordination with the others to complete the collection, sending, monitoring and early warning functions of human physiological data. The data processing center of the whole system uses a control unit for data control and transmission, requires a strong computing power, can quickly process a variety of requests, and needs to provide enough communication interfaces for a variety of peripheral equipment connections. Since the terminal collects the physiological data of the human body, it must transmit in real time and reliably, which requires that the information-receiving and information-sending unit provide a safe and reliable communication process. The most important feature of the data acquisition unit is that it requires high sensitivity, so that it can quickly reflect the changes in the body and convert them into visual data. The early warning part can act as a reminder, when the data exceed the set range, the buzzer immediately sends out an alarm. The display module displays the data collected in real time, so that the detection data can be viewed at any time.

#### Control unit

**Fig. 3:**
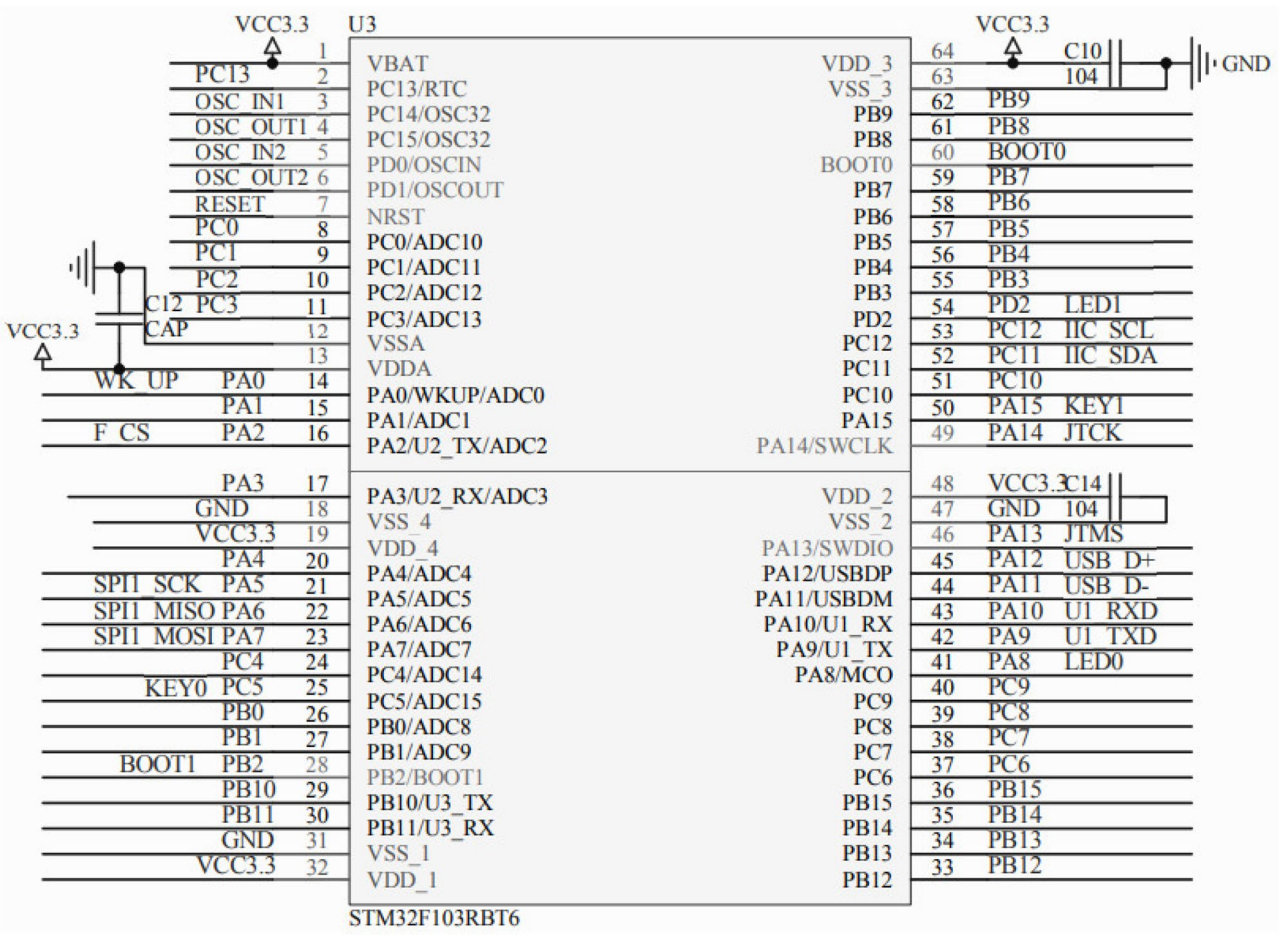
Main controller circuit of STM32F103

The control chip is the smallest system board of the STM32F103 series. The controller has a 32-bit bit width, a 72 MHz frequency, 48KB of static RAM, 256KB of flash memory, multiple timers with different functions, multiple I2C interfaces, UART/USART data transmission interfaces, and a variety of peripheral devices such as the ADC and DMA in the smallest system. As the ADC conversion frequency, I2C clock frequency, PWM wave output and other clock control functions are involved in the design, the minimal system’s external clock circuit is necessary in the hardware design to provide the system with more accurate and reliable clock output. The main controller circuit of STM32F103 is shown in Fig. 3.

#### Communication and positioning module

**Fig. 4:**
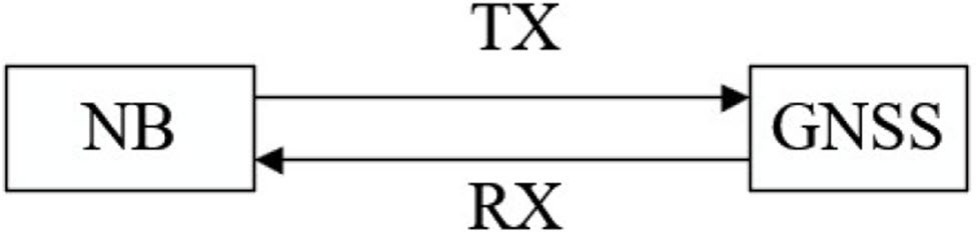
NB and GNSS communication wiring

The communication and GPS module is an integrated BC20 module that integrates NB-IoT and GNSS functions, supports GPS positioning technology, supports AGPS positioning assistance technology, has low power consumption, anti-interference, and high precision characteristics, and also provides a variety of pin settings in different frequency bands. The packaging design integrates a GSM module and a GPRS module, with a network service protocol stack embedded internally. While performing mobile communications, it can also rapidly and accurately acquire latitude and longitude information from the GNSS. This approach provides users with an efficient GPS data acquisition experience at a very low cost. In addition, BC20 also supports OneNET, Baidu Cloud, Tencent cloud and other cloud platforms, which are widely used by developers for the development of various IoT devices. The NB and GNSS communication wiring is shown in Fig. 4.

**Fig. 5:**
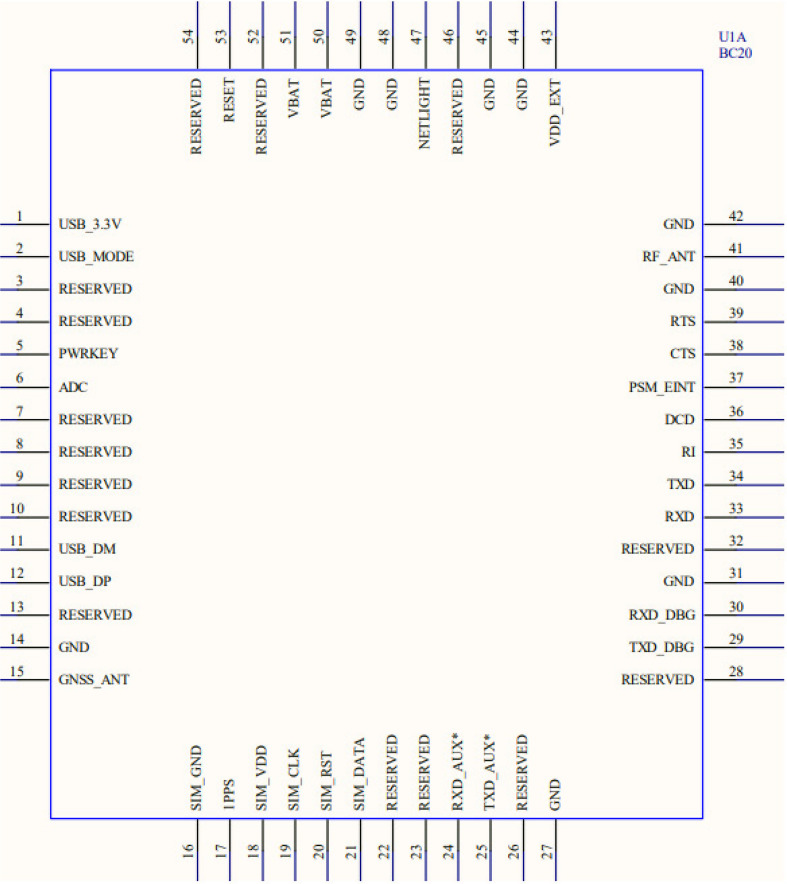
BC20 core chip interface

The BC20 module has NB and GNSS functions, so the SIM card interface and GNSS RF interface are provided in its hardware and the BC20 core chip interface is shown in Fig. 5.

#### Terminal sensor

##### Heart rate and blood oxygen module design

A MAX30102 high sensitivity pulse oximeter and heart rate sensor are selected in this module. It integrates an 18-bit ADC chip and 32-bit memory.

**Fig. 6:**
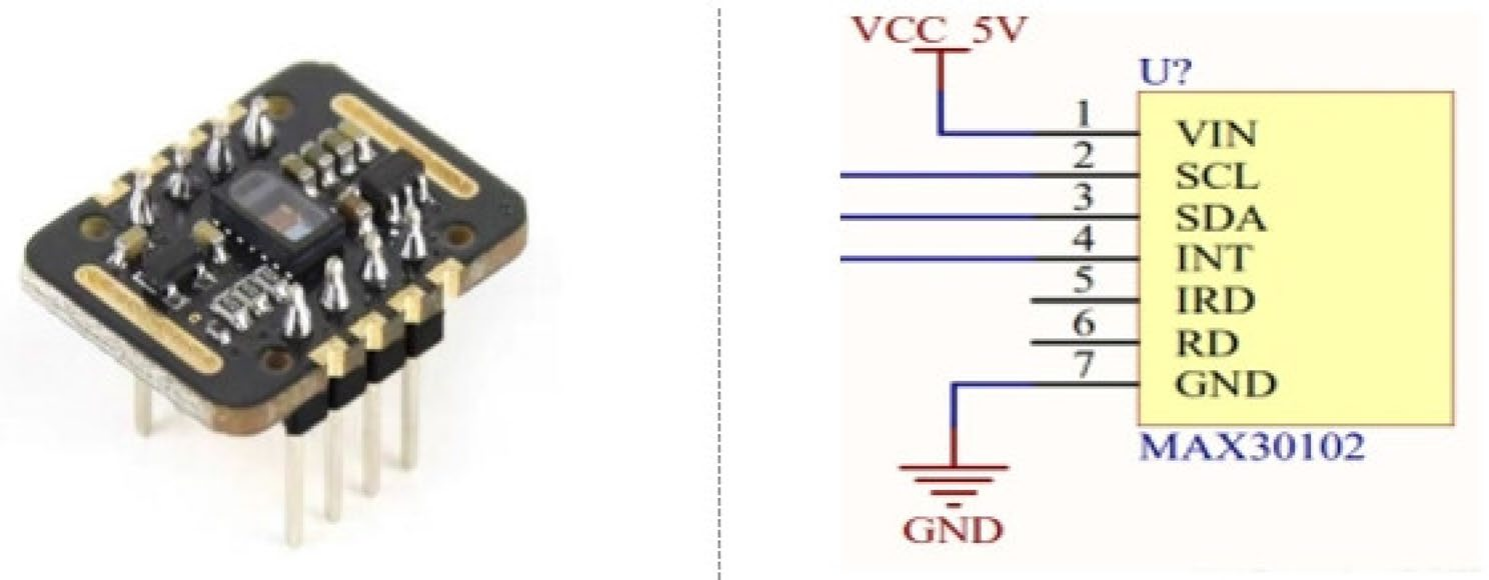
Heart rate and blood oxygen sensors and their interface circuits

The MAX30102 heart rate and blood oxygen sensor uses a dual-bus I2C communication bus. It utilizes the PB6 and PB7 pins of the STM32F103RCT6 control chip to act as the clock line and data line, controlling MAX30102 to implement specific read/write timing sequences and configure and read internal registers. After it powers on, the INT interface continuously outputs a high level. When it detects the skin approaching, it generates a low-level signal, serving as an interrupt source for the microcontroller. After the returned data are processed, accurate heart rates and blood oxygen values are obtained. The heart rate and blood oxygen sensors and their interface circuits are shown in Fig. 6.

##### Blood pressure module design

**Fig. 7:**
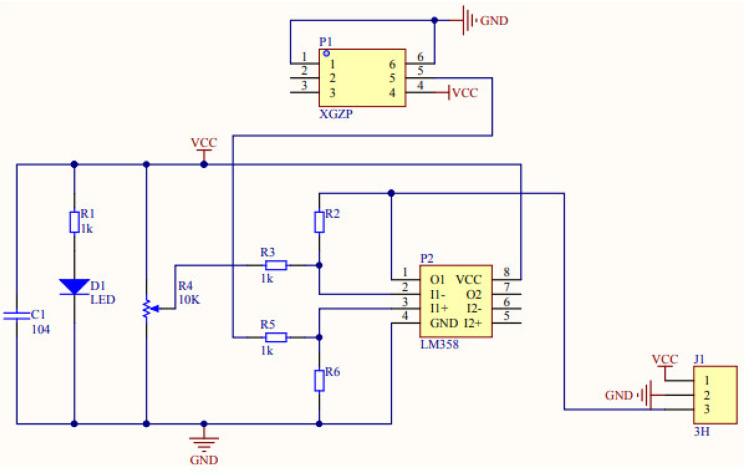
Circuit diagram of MPS20 pressure sensor

The MPS20 pressure sensor is selected to simulate the detection of blood pressure in the blood pressure module. The circuit diagram of the MPS20 pressure sensor is shown in Fig. 7. An LM358 dual operational amplifier is added to the circuit and can be used for internal frequency compensation and linear amplification of the internal voltage to output a linear voltage signal related to pressure. The AO output is subsequently fed into one of the A/D analog to digital conversion ports PA0 of the STM32F103RCT6 control chip.

##### Design of the temperature measurement module

**Fig. 8:**
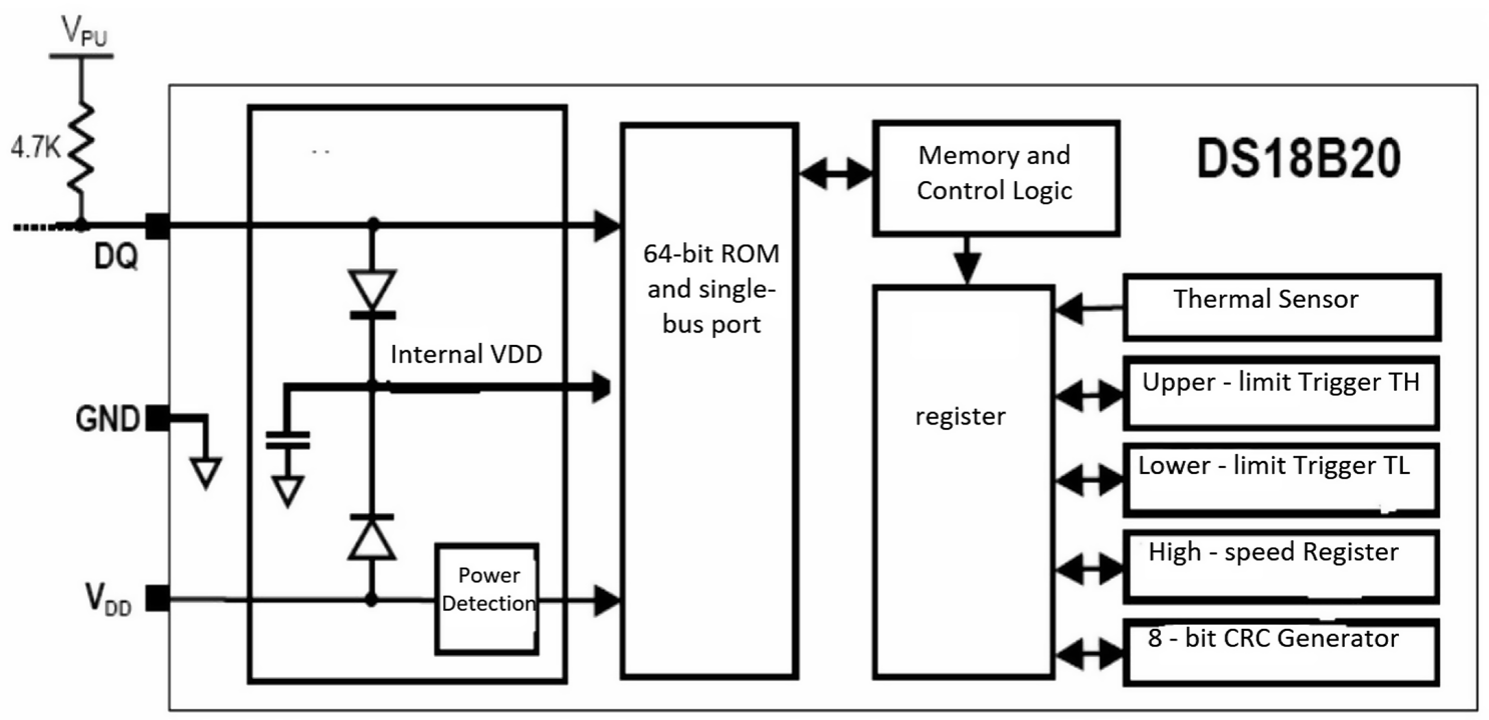
Internal structure of DS18B20 sensor

As a highly sensitive temperature measurement element, DS18B20 is the best choice for the temperature measurement module. The internal structure of the DS18B20 sensor is shown in Fig. 8. The DS18B20 internal structure is shown below. Table 2 shows the ROM structure of DS18B20 and Table 3 shows the DS18B20Common command list.

**Table 2:** ROM structure of DS18B20

8-digit CRC number	48-bit serial number	8-bit product family code

**Table 3:** DS18B20Common command list

Name of the instruction	Instruction code	8-bit command function
Read ROM	33 H	This command can be used to read the DS18B20 64-bit ROM code back to the controller, but only when a single sensor is on the bus
Match ROM	55 H	This instruction initiates DS18B20 on the matching bus, after which the ROM address must be sent for matching
Skip ROM	CCH	This instruction can skip the matching ROM encoding step and send the temperature conversion instruction directly, but it is only suitable for a single sensor on the bus
Temperature change	44 H	This instruction causes DS18B20 to perform a temperature conversion operation and stores the conversion result in a scratchpad
Read the register	BEH	The contents of 9 bytes of internal RAM
are read Write scratchpads	4EH	This instruction is used to write the starting instruction for the upper and lower temperature limits of the register, after which two data points must be sent to set the threshold
Read the power supply mode	B4H	The power supply mode of the sensor is read, the controller receives the reply signal “0” when the parasitic power supply is supplied, and reply signal “1” when the external power supply is connected

**Fig. 9:**
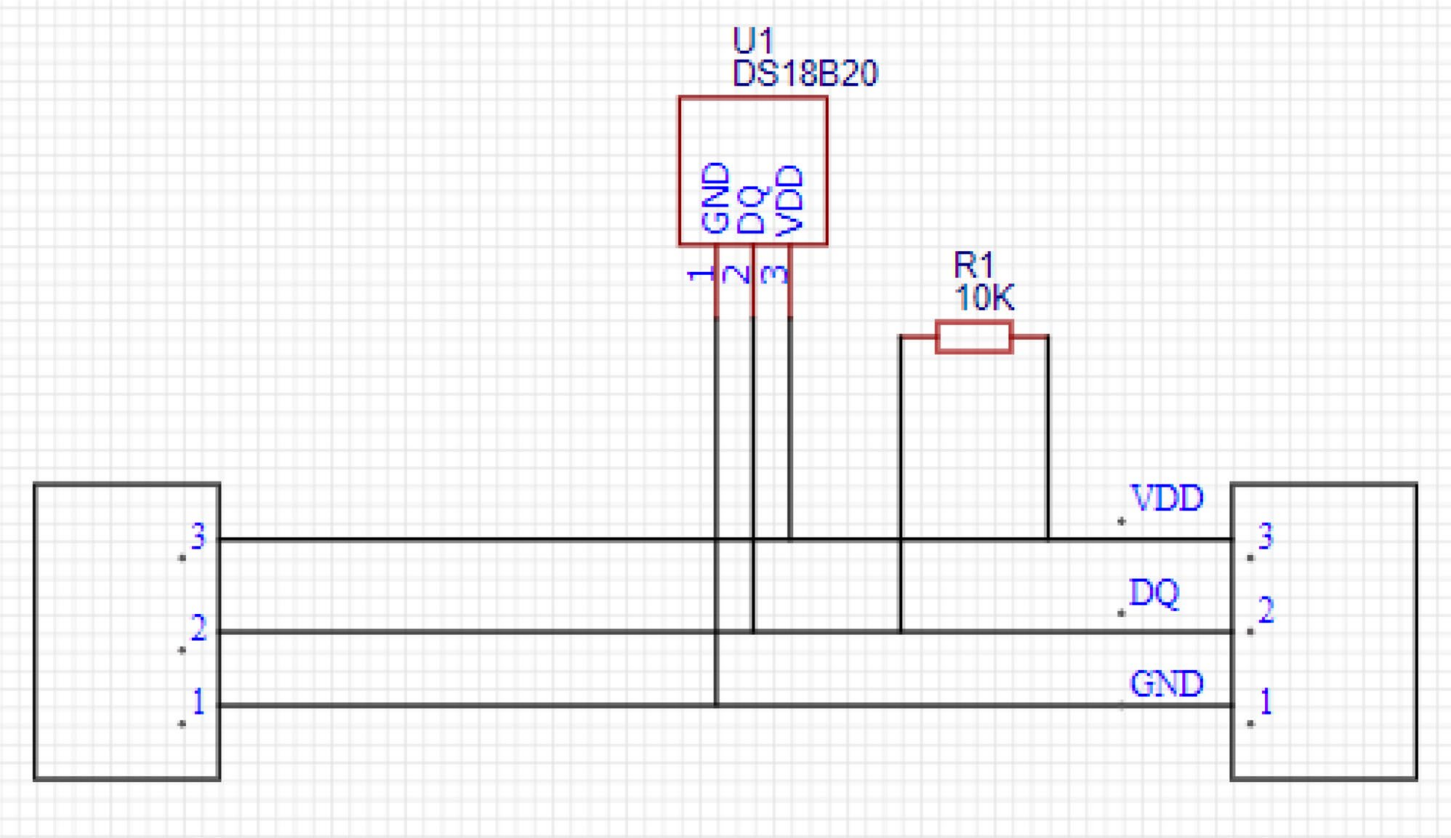
Interface circuit of DS18B20

When the external device is connected to the single-wire port through the drain of the triode, the DS18B20 needs a resistor in series with a weak power supply to form a pull-up resistor so that the sensor has sufficient current to maintain the normal operation of the internal components. During communication with the control chip, the DQ pin is connected to the PA7 pin on the STM30F103RCT6 control chip. The control chip relies on the address within the sequence number to locate the corresponding temperature sensor and read its real-time temperature value. Figure 9 shows the hardware interface circuit of DS18B20.

#### Early warning module

An early warning is a module used by most data monitoring devices, and can monitor abnormal data. In this design, a passive buzzer is selected as the alarm.

**Fig. 10:**
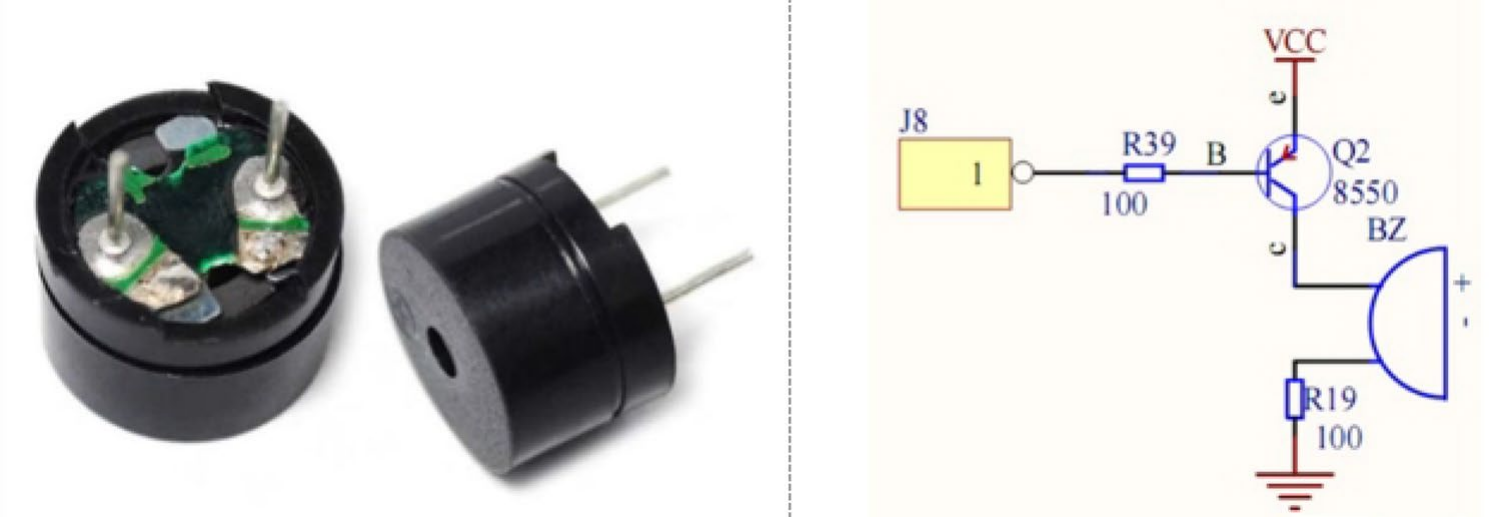
The overall appearance of the passive buzzer and its circuit structure

Figure 10 shows the overall appearance of the passive buzzer and its circuit structure. Owing to the limited current output of the GPIO port of STM32, which is only at the microamp level, the normal operation of the buzzer cannot be supported. Therefore, in the circuit design, a 8550 triode is employed to amplify the drive current. In the system, the PA15 pin of the STM32F03RCT6 control chip is connected to base B. When the heart rate, blood oxygen, blood pressure and body temperature data exceed the set threshold, the PA15 pin of the control chip outputs the PWM pulse signal to drive the buzzer to issue an alarm.

#### Display module

**Fig. 11:**
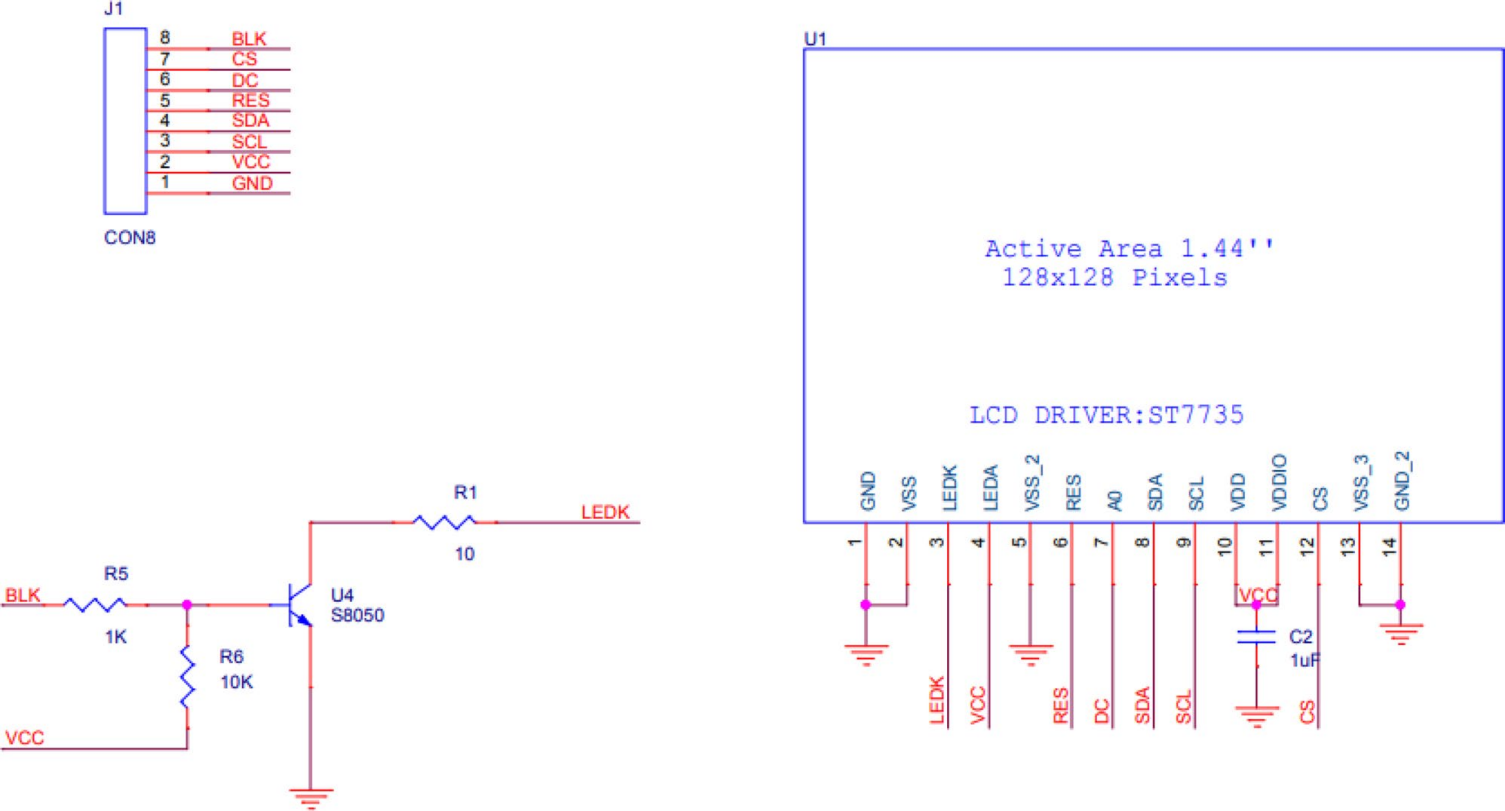
Circuit of the TFT LCD

The display module uses a 1.44-inch TFT LCD screen, that has a dot matrix size of 128 × 128, providing sufficient pixel space for display. Figure 11 shows the circuit of the TFT LCD. The figure shows that its data transmission uses the SPI communication protocol, a BLK pin is provided for backlight control, a CS pin is provided for chip selection, a DC pin is provided for reading and writing registers, and an RES pin is provided for restarting the screen. The PB4, PB5, PB12, PB13, PB14, and PB15 pins of the STM32F103RCT6 control chip are connected with the BLK, DC, RES, CS, SCL and SDA pins of the TFT LCD screen respectively to control the content required by the display. Additionally, since the display relies on the brightness of the pixels to render text, precise control of these pixels is of paramount importance. Therefore, in the design, a PC is used in LCD module software to display the Chinese characters required for module processing.

### Program design and terminal equipment

#### STM32 main program design

**Fig. 12:**
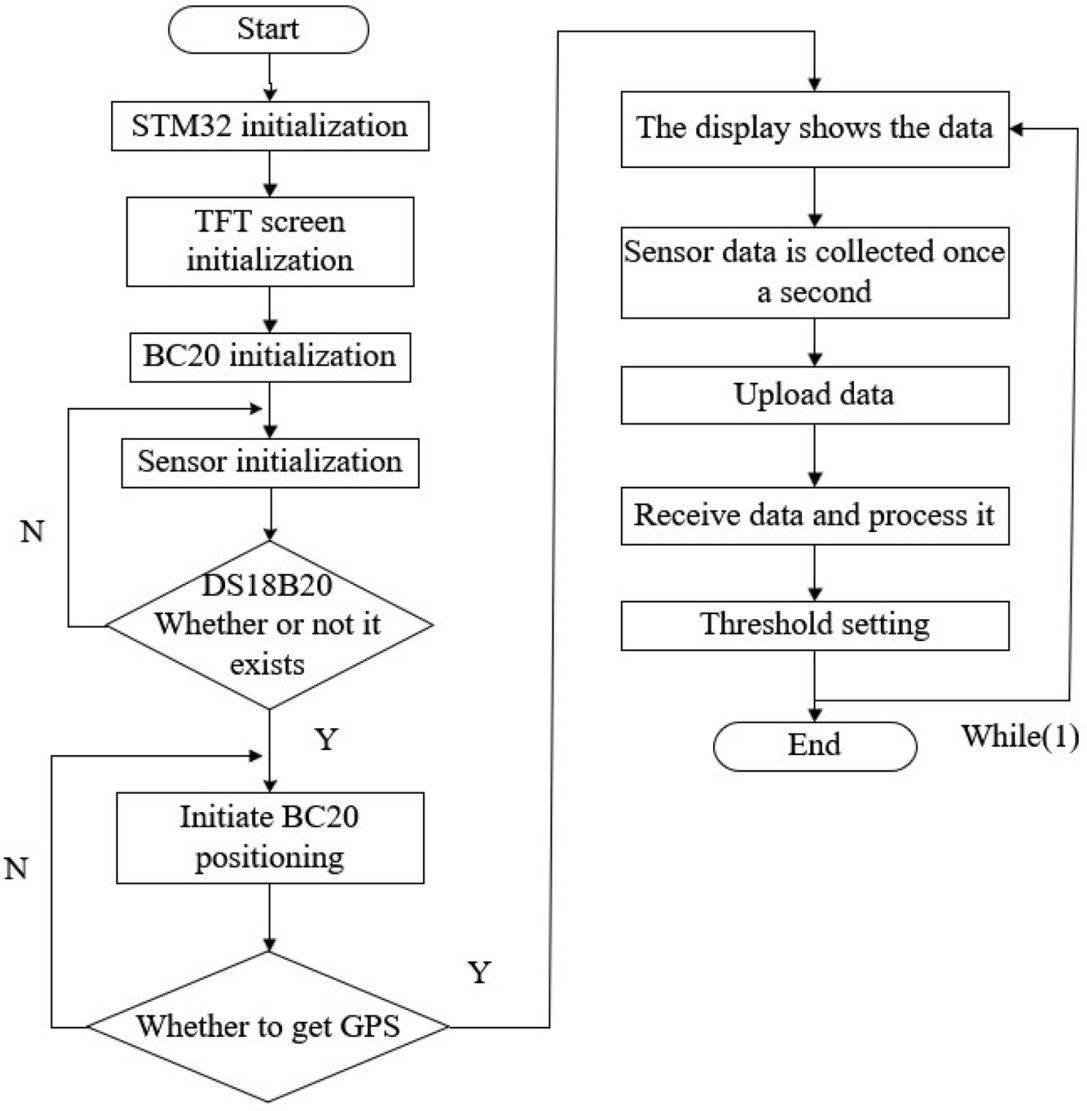
Flow chart of the STM32F103RCT6 main handler

The flow chart of the STM32F103RCT6 main handler is shown in Fig. 12. After the program starts running, the STM32 GPIO port, ADC, I2C and timer functions, MAX30102 heart rate blood oxygen sensor and DS18B20 temperature sensor are initialized. Then, the BC20 module is controlled to connect to the MQTT server, and the latitude and longitude information of the device is obtained. The heart rate, blood oxygen, blood pressure and temperature sensor data are collected at one-second intervals, and the collected various processed data are uploaded at two-second intervals. Since the mobile terminal can set the threshold value at any time after it connects to the server, the communication module may receive information from the mobile terminal at any time. Therefore, in the main program, after the BC20 is connected to the server, it checks whether the BC20 module has received the message every millisecond.

#### Communication and GPS module program design

**Fig. 13:**
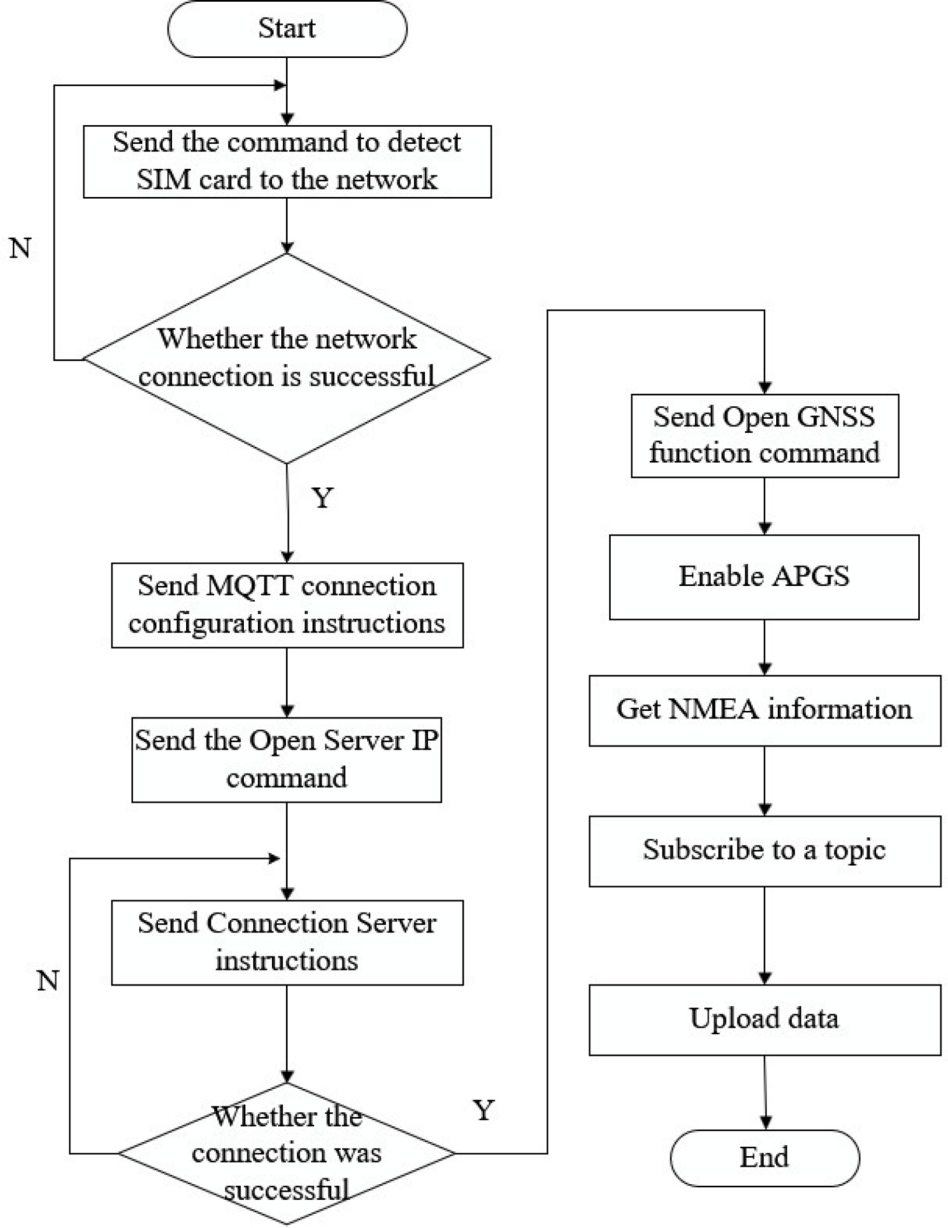
Flow chart of the communication and localization procedure

The flow chart of the communication and localization procedure is shown in Fig. 13. In the whole communication process, the SIM card signal quality and network access detection is carried out first. If the signal quality of the SIM card is too poor or the network access is not successful, the card cannot be connected to the network and the subsequent operation cannot be carried out. Therefore, the instructions must be resent, or the device needs to be transferred to a place with better signal quality. Second, the parameters such as the connection heartbeat and timeout time of the MQTT client must be set when the configuration instruction is sent. Subsequently, an instruction to open the destination IP as the server must be sent to bind the server IP. Then, connection to the server is established, after the subsequent can steps be carried out. The last step is to turn on the GNSS function and enable the APGS to obtain the accurate latitude and longitude information, then subscribe to the required topics and upload the obtained data to the server.

In the program design of the communication and GPS module, the AT instruction structure is designed to save the instructions sent and received. Within this structure, the timeout period for command transmission, the status, and the number of times the command is resent in the case of failure are also included. The status of the instruction after sending is an enumeration type. The members of the enumeration are SUCCESS$\_$REC, TIME$\_$OUT, NO$\_$REC and ERROR$\_$REC, which indicate that the instructions are send successfully, sending timeout occurs, the response is not received and sending failure occurs, respectively.

In the design of a program that can send instructions, the state machine and the current instruction value are also designed. The members of the state machine enumeration type are NB$\_$IDILE, NB$\_$SEND and NB$\_$WAIT, which represent the current state of the STM32 controller chip. NB$\_$IDILE means that the current microprocessor is in the idle state. That is, no AT instruction is sent, and no reply is waiting for the BC20 module. NB$\_$SEND indicates that the current microprocessor is in the state of sending commands, and NB$\_$WAIT indicates that the current microprocessor has sent an AT instruction and is waiting for a response from the BC20 module. The enumeration type elements of the current instruction value are AT$\_$CSQ, AT$\_$CGPADDR, AT$\_$QMTCFG, AT$\_$QMTOPEN, AT$\_$QMTCONN, AT$\_$QMTSUB, and AT$\_$QMTPUB, which indicate the instructions sent by the current microprocessor. The enumerator type is incremented to monitor the AT instruction.

#### Heart rate and blood oxygen test program design

**Fig. 14:**
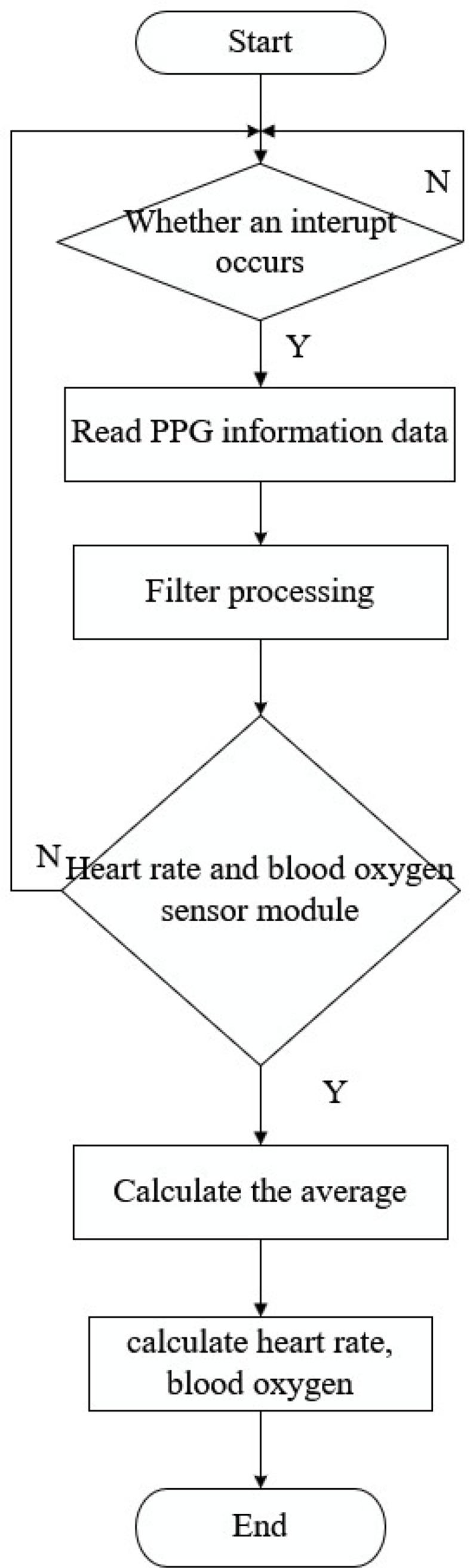
Heart rate blood oxygen module program design flow chart

In the system, the MAX30102 heart rate blood oxygen sensor starts to run after the power is turned on. When the skin approaches the sensor and is subsequently detected, the MAX30102 interrupt pin generates a low level signal, which is the external interrupt source of STM32F103RCT6. When the interrupt signal is generated, the PPG signal data in the MAX30102 FIFO register are read, and the data are filtered. Then, according to the set threshold, whether the acquisition should be started again is determined to exclude the case of false contact. Finally, the average value of multiple data points is used to calculate the heart rate and blood oxygen, which makes the detection results more accurate. The main code for heart rate blood oxygen acquisition is shown in Fig. 14.

#### Program design for blood pressure detection

The data captured by the MPS20 pressure sensor are a continuous analog signal that requires conversion into digital format using the STM32F103RCT6 minimum system and its built-in AD conversion module. In this system, the adopted frequency of the ADC is set to 8 MHz, the sampling period is 1.5 clock cycles, and 12.5 conversion clock cycles are added, thus, it takes 14 clock cycles are needed to complete a digital-to-analog conversion. By calculating 14/14 MHz, AD conversion can be completed every 1 microsecond. The resulting voltage is a series of values that are linear with respect to pressure and can be converted to the final pressure value by the algorithm described above.

#### Human body temperature detection program design

The DS18B20 temperature sensor is connected to the STM32 control chip through a single bus. Each time the sensor is driven for temperature conversion, the following steps are needed: initialization, sending of ROM operation commands, and sending of memory operation commands.

Initialization: The host generates a reset pulse by pulling down a single wire above 480 $\mu$ s, and then releases the wire to enter Rx receive mode. When the host releases the bus, a rising edge pulse is generated. After the DS18B20 detects the rising edge, it delays by 15 - 60 $\mu$ s and generates the response pulse by pulling the bus down by 60 - 240 $\mu$ s. The receipt of the response pulse from the slave by the master, indicates that a single wire device is online. At this point, initialization is complete.

Send the ROM operation command: When the host detects the response pulse, it can initiate the ROM operation command. The ROM operation command is used to read the 64-bit ROM sequence number of the sensor on the bus, and the corresponding DS18B20 sensor can be addressed with the sequence number.

Send memory operation command: The memory operation command can be used only after the ROM operation command is successfully executed. The designer can read the contents of the scratcher and configure the configuration registers of DS18B20 through the memory manipulation instructions. After the data are read and removed, they can be converted into temperature data by program code.

## Results

**Fig. 15:**
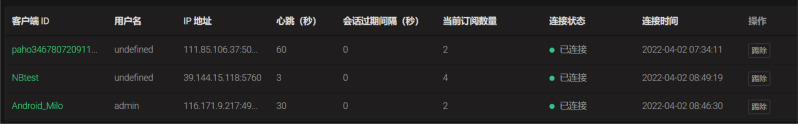
MQTT server background management interface

**Fig. 16:**
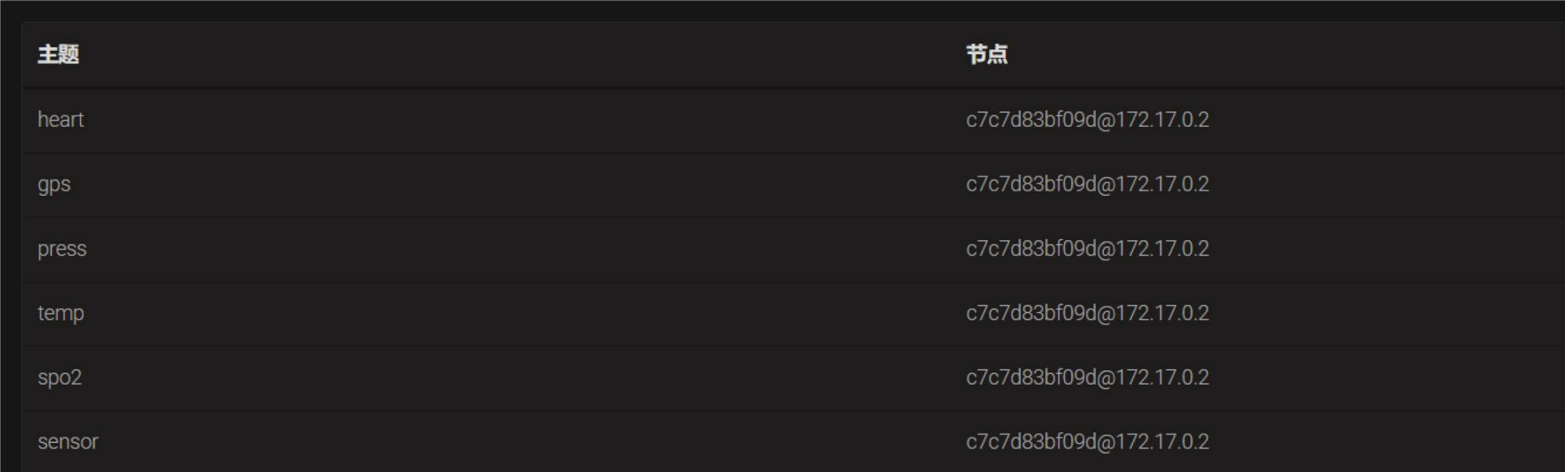
Client topic subscription graph

**Fig. 17:**
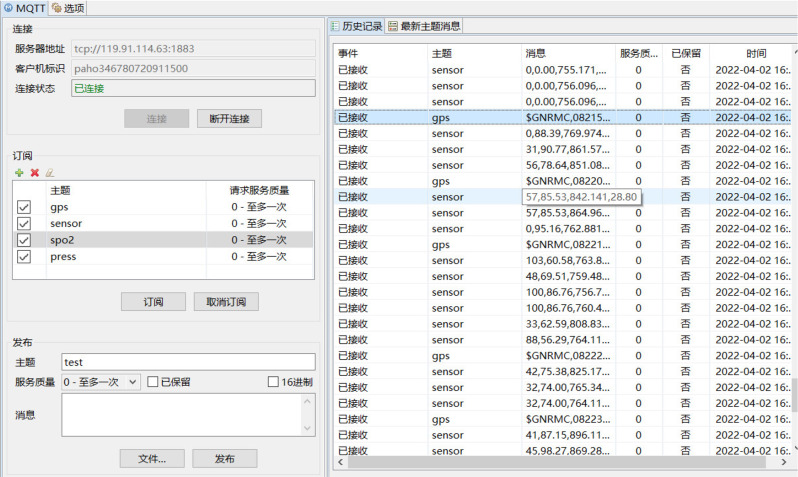
Paho software information reception

First, we test the connectivity between the monitoring device and the server. The client device named NBtest is successfully connected to the MQTT server as shown in Fig. 15. After connecting to the MQTT server, the client directly subscribes to the topic of the sensor threshold and uploads the real-time data acquired by the sensor to the server. As shown in Fig. 16, the client topic subscription graph shows that the topic subscription is successful. The Paho software information reception diagram is shown in Fig. 17, which shows that the hardware device is successfully communicated and can send real-time data to another client.

Next, after the system hardware and software deployment are connected, the operation of each sensor is tested. As shown in Table 4, statistics are collected for the same person’s heart rate, blood oxygen and body temperature, as well as simulated blood pressure data. The heart rate, blood pressure, blood oxygen, and body temperature parameters collected by the sensors are basically stable. This test ensures that the design and implementation of our health collection system are successful and can communicate with the server normally.

**Table 4:** Collected vital physiological parameter data

Data name	Heart rate (beats/min)	Blood oxygen ($\%$)	Blood pressure (mmHg)	Body temperature($^{\circ}$C)
1	71	91.90	755.17	28.10
2	71	99.34	756.40	28.10
3	71	98.86	756.40	28.10
4	68	99.84	756.71	28.10
5	100	98.97	757.71	28.10
6	81	99.93	755.78	28.20
7	78	99.91	756.71	28.20
8	73	99.64	758.25	28.20
9	81	99.42	756.71	28.30
10	81	99.42	757.02	28.30

**Fig. 18:**
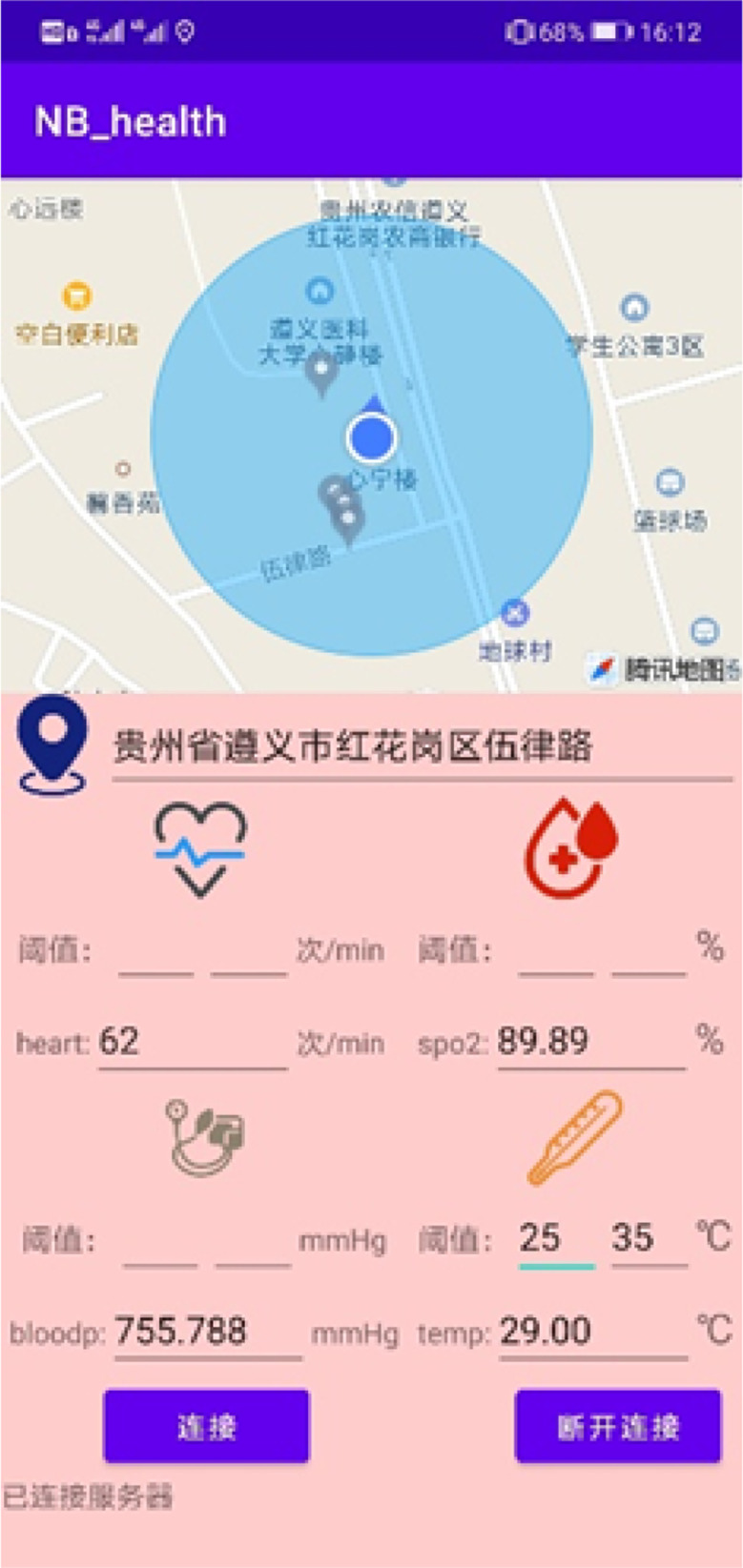
Mobile APP positioning test diagram

Finally, we design an Android application that displays health data, sets health parameter thresholds, and tracks real-time locations. The location tracking feature can help caregivers or family members query the location information of the monitored person through the Android application. Figure 18 shows the interface of the mobile application, from which the app successfully displays the monitored person’s location, heart rate, blood pressure, blood oxygen, and body temperature parameters, as well as the corresponding health parameter threshold setting function. The system allows users to customize the threshold of physiological parameters, forming a user-adaptive health monitoring system and providing timely warnings in abnormal situations.

## Discussion

The test results verified that the system’s hardware integration and software implementation effectively realized real-time acquisition, remote transmission, abnormal early warning, and navigation positioning functions of vital physiological parameters. This confirms that the developed system meets the design requirements for portable remote health monitoring for portable and remote health monitoring of elderly patients in remote areas. Compared with existing remote monitoring studies, this study combines STM32 microcontroller, multi-sensor modules, MQTT communication protocol, and Android application to form a low-cost, low-power, and easy-to-operate solution, which better addresses the problems of large size, high cost, and short communication distance in traditional devices, and provides reliable daily health monitoring and emergency response support for elderly populations in resource-limited regions.

The limitations of this study include that the blood pressure detection adopts simulated sensing rather than clinical-grade measurement, and the system only carries out laboratory functional tests without long-term field verification in complex remote environments. Future work will focus on upgrading medical-grade sensors to improve detection accuracy, optimizing communication stability for weak network conditions, and integrating intelligent analysis algorithms to enhance health risk prediction, so as to further promote the clinical application and promotion of the system.

## Conclusions

This study presents the development of a remote vital health monitoring system based on microcontroller technology, wireless communication technology, the MQTT protocol, sensing technology, and Android application development. The system exhibits characteristics such as cost-effectiveness, convenience, and real-time monitoring. In addition, the system is an android-based health monitoring application that supports real-time monitoring of several key physiological parameters, including heart rate, blood oxygen, blood pressure, and body temperature. Moreover, the application we have developed has a wide range of use scenarios. It is suitable not only for elderly patients in remote areas with limited medical resources but also for disabled individuals with mobility issues and elderly patients with chronic conditions. The system supports user-defined thresholds for physiological parameters, enabling the formation of a personalized health monitoring system that promptly alerts caregivers in the event of abnormal conditions. The system was tested, and its GPS positioning function, communication function, vital physiological parameter monitoring function, real-time monitoring function, and early warning function all work properly. Additionally, the outputs of the data from various sensors are stable. The monitoring system can measure vital physiological parameters, providing positioning information, and timely alerts. This enables caregivers to promptly grasp the health status of the monitored person, resulting in the intended outcome.

## Data Availability

No datasets were generated or analysed during the current study.
